# Antibacterial activities of selected Cameroonian spices and their synergistic effects with antibiotics against multidrug-resistant phenotypes

**DOI:** 10.1186/1472-6882-11-104

**Published:** 2011-11-01

**Authors:** Aimé G Fankam, Victor Kuete, Igor K Voukeng, Jules R Kuiate, Jean-Marie Pages

**Affiliations:** 1 Department of Biochemistry, Faculty of science, University of Dschang, Cameroon; 2 Transporteurs Membranaires, Chimiorésistance et Drug Design, UMR-MD1, IFR 88, UFRs de Médecine et de Pharmacie, Marseille, France

## Abstract

**Background:**

The emergence of multi-drug resistant (MDR) phenotypes is a major public health problem today in the treatment of bacterial infections. The present study was designed to evaluate the antibacterial activities of the methanol extracts of eleven Cameroonian spices on a panel of twenty nine Gram negative bacteria including MDR strains.

**Methods:**

The phytochemical analysis of the extracts was carried out by standard tests meanwhile the liquid micro-broth dilution was used for all antimicrobial assays.

**Results:**

Phytochemical analysis showed the presence of alkaloids, phenols and tannins in all plants extracts. The results of the antibacterial assays indicated that all tested extracts exert antibacterial activities, with the minimum inhibitory concentration (MIC) values varying from 32 to 1024 μg/ml. The extracts from *Dichrostachys glomerata, Beilschmiedia cinnamomea*, *Aframomum citratum*, *Piper capense*, *Echinops giganteus*, *Fagara xanthoxyloïdes *and *Olax subscorpioïdea *were the most active. In the presence of efflux pump inhibitor, PAßN, the activity of the extract from *D. glomerata *significantly increased on 69.2% of the tested MDR bacteria. At MIC/5, synergistic effects were noted with the extract of *D. glomerata *on 75% of the tested bacteria for chloramphenicol (CHL), tetracycline (TET) and norfloxacin (NOR). With *B. cinnamomea *synergy were observed on 62.5% of the studied MDR bacteria with CHL, cefepime (FEP), NOR and ciprofloxacin (CIP) and 75% with erythromycin (ERY).

**Conclusion:**

The overall results provide information for the possible use of the studied extracts of the spices in the control of bacterial infections involving MDR phenotypes.

## Background

The emergence of MDR phenotypes is a major public health problem today in the treatment of bacterial infections. The multi-drug resistance of Gram negative bacteria is a major cause of morbidity and mortality in health care services [1]. The activation of bacterial efflux pumps also plays an important role in the appearance of resistance to antibiotics [2]. The real challenge for scientists worldwide today, is to continuously find new drugs to combat resistant microorganisms, or compounds which are able to inhibit the resistance mechanisms of pathogens, therefore restoring the activity of antibiotics. Medicinal plants are rich in compounds which may be potential natural drugs and serve as alternative, less expensive and safe antimicrobials for the treatment of common ailments. Plant drugs are widely used in Africa for the treatment of many ailments and constitute the first health recourse for about 80% of the population [3]. A number of pharmaceutical products in current use worldwide are derived from plants [4]. In Cameroon, many medicinal plants including spices are used as herbal medicines. The present work was therefore designed to investigate the antibacterial potential against MDR bacteria of some of the commonly used medicinal spices in Cameroon such as *Fagara xantoxyloides *Watern.*, Dichrostachys glomerata *(Forsk) Chuov, *Olax subscorpioïdea *Oliv.*, Solanum melongeua *L. Var inerme D.C Hiern, *Piper capense *Lin.f, *Xylopia aethiopica *Dunal A. Rich., *Aframomum citratum *(Pereira). Schum, *Scorodophloeus zenkeri *Harms.*, Beilschmiedia cinnamomea *(Stapf) Robyns & Wilczek, *Echinops giganteus *A. Rich and *Mondia whitei *(Hook F). Skell. This study was also extended to the evaluation of the potencies of the above plant extracts to increase the activity of some antibiotics on MDR bacteria. The role of bacterial efflux pumps in resistance to the extracts was also studied.

## Methods

### Plant materials and extraction

The eleven edible spices used in this work were purchased from Dschang local market, West Region of Cameroon in January 2010. The collected spices materials were: the fruits of *Fagara xanthoxyloides, Dichrostachys glomerata, Olax subscorpioïdea, Solanum melongeua, Piper capense *and *Xylopia aethiopica*, the bark of *Aframomum citratum, Scorodophloeus zenkeri, Beilschmiedia cinnamomea *and the roots of *Echinops giganteus *and *Mondia whitei*. The plants were identified by Mr. Fulbert Tadjouteu of the National herbarium (Yaoundé, Cameroon) where voucher specimens were deposited under the reference numbers (Table 1).

**Table 1:** Spices used in the present study and evidence of their activities.

Spice samples (Family)	**Herbarium Voucher number**^**a**^	Part used	Bioactive (or potentially active) compounds^b ^and screened activity^c ^for crude plant extract
*Fagara xanthozyloïdes* Watern. (Rutaceae)	21793/HNC/SRF	Fruits	Antimicrobial activity of essential oil [S: Ec, Bc, Bs, Af, Kp, Sa, Sf [19]; Cytotoxicity of fruits crude methanol extract [weak activity on leukemia CCRF-CEM and CEM/ADR5000 cells, and pancreatic MiaPaCa-2 cell lines] [27]
*Dichrostachys glomerata* *(*Forsk) chuov (Mimosaceae)	15220/SRF-Cam	Bark, fruits	Cytotoxicity of roots crude methanol extract [weak activity on leukemia CCRF-CEM and CEM/ADR5000 cells, and pancreatic MiaPaCa-2 cell lines][27]
*Aframomum citratum* (Pereira). Schum (Zingiberaceae)	37736/SRF-Cam	Leaves, fruits	Cytotoxicity of leaves crude methanol extract [weak activity on leukemia CCRF-CEM and CEM/ADR5000 cells, and pancreatic MiaPaCa-2 cell lines] [27]
*Beilschmiedia cinnamomea *(Stapf) Robyns & Wilczek (Lauraceae)	6933/SRF-Cam	Roots	/
*Echinops giganteus* A. Rich. (Asteraceae)	23647/SRF-Cam	Rhizomes	Antimicrobial [lupeol sitosteryl; β-D-glucopyranoside] [28-31]; Cytotoxicity of rhizome crude methanol extract [Significant activity with IC_50 _values of 6.68; 7.96 and 9.84 μg/ml respectively on leukemia CCRF-CEM cells, CEM/5000 cells and pancreatic MiaPaCa-2 cell lines] [27]
*Mondia whitei* (Hook F). Skell. (Periplocaceae)	42920/HNC	Fruits	Reproduction system [Roots water extract (400 mg/kg/day) for 55 days caused testicular lesions resulting in the cessation of spermatogenesis, degenerative changes in the somniferous tubules and epididymides in rats] [32]
*Olax subscorpioidea* Oliv. (Olacaceae)	3528/SRFK	Seeds	Antibacterial and cytotoxic against *Artemia salina *[Santalbic acid] [33,34]; Cytotoxicity of leaves crude methanol extract on cancer cells [weak activity on leukemia CCRF-CEM and pancreatic MiaPaCa-2 cell lines and significant activity with IC_50 _of 10.65 μg/ml on CEM/ADR5000 cells] [27]
*Solanum melongena *L.Var inerme D.C Hiern. (Solanaceae)	22615/SRFC	Fruits	Antimicrobial activity of methanol, dichloromethane and petrol ether extracts of the fruits: [Q: Tm, Tr, Tt, Ca et Tb [35]
*Piper capense* Lin.f (Piperaceae)	7650/SRF-Cam	Fruits	Insecticidal [N-isobutyl-ll-(3, 4-methylenedioxyphenyl)-2*E*, 4*E*, 10*E*-undecatrienamide; N-pyrrolidyl-12-(3, 4-methylene-dioxyphenyl)-2*E*, 4*E*, 9*E*, 11*Z*-dodecatetraenamide; N-isobutyl-13-(3, 4-methylenedioxyphenyl)-2*E*, 4*E*, 12*E*-tridecatrienamide; N-isobutyl-2*E*, 4*E*-decadienamide; N-isobutyl-2*E*, 4*E*-dodecadienamide] [36]; Cytotoxicity of fruit crude methanol extract [Significant activity with IC_50 _values of 7.02; 6.56 and 8.92 μg/ml respectively on leukemia CCRF-CEM cells, CEM/5000 cells and pancreatic MiaPaCa-2 cell lines] [27]
*Xylopia aethiopica *(Dunal) A. Rich. (Annonaceae)	16419/SRF-Cam	Bark, leaves, roots, seeds	Antimicrobial [volatile oil of seeds] [19]; Antioxidant [volatile oil of seeds] [37]; Cytotoxicity of seeds crude methanol extract [Significant activity with IC_50 _values of 3.91; 7.4 and 6.86 μg/ml respectively on leukemia CCRF-CEM cells, CEM/5000 cells and pancreatic MiaPaCa-2 cell lines] [27]
*Scorodophloeus zenkeri* Harms. (Caesalpinaceae)	44803/SRF-Cam	Bark	Antimicrobial: [2, 4, 5, 7-Tetrathiaoctane; 2, 4, 5, 6, 8-pentathianonane; 2, 3, 4, 6, 8-pentathianonane; 2, 3, 5, 6, 8, 10-hexathiaundecane; 2, 3, 5-trithiahexane 5-oxide; 2, 4, 5, 7-tetrathiaoctane 2-oxide; 2, 3, 5, 7-tetrathiaoctane 3, 3-dioxide; 2, 3, 5-trithiahexane 3, 3-dioxide [38]; Cytotoxicity of bark crude methanol extract on cancer cells [weak activity on leukemia CCRF-CEM and pancreatic MiaPaCa-2 cell lines and significant activity with IC_50 _of 10.65 μg/ml on CEM/ADR5000 cells] [27]

^a^(HNC): Cameroon National Herbarium; (SRF): Société des reserves forestières; Cam: Cameroon; ^b^(/): Not reported

^c^[Screened activity: significant (S: CMI < 100 μg/ml), moderate (M: 100 < CMI ≤ 625 μg/ml), Weak (W: CMI > 625 μg/ml) Q: Qualitative activity based on the determination of inhibition zone [11,12]. *Tm: Trichophyton mentagophytes; Tr: Trichophyton rubrum; Tt: Trichophyton tonsurans Tb: Trichosporon beigelii; Ca: Candida albicans; Ck: Candida krusei; Af: Aspergillus flavus; Bc: Bacillus cereus; Bs: Bacillus subtilis; Ec: Escherichia coli; Kp: Klebsiella pneumoniae; Sa: Staphylococcus aureus; Sf: Streptococcus faecalis*.

The air dried and powdered sample (1 kg) from each spice was extracted with methanol (MeOH) for 48 h at room temperature. The extract was then concentrated under reduced pressure to give residues which constituted the crude extracts. They were then kept at 4°C until further use.

### Preliminary phytochemical investigations

The major classes of secondary metabolites; alkaloids, anthocyanins, anthraquinones, flavonoids, phenols, saponins, tannins, steroids and triterpenes were screened according to the common phytochemical methods described by Harborne [5] with some modifications. Briefly, for alkaloids (5 mg plant extract in 10 ml methanol); a portion of 2 ml extract + 1% HCl + steam, 1 ml filtrate + 6 drops of Mayor's reagents/Wagner's reagent/Dragendroff reagent; creamish precipitate/brownish-red precipitate/orange precipitate indicated the presence of respective alkaloids. For tannins (5 mg plant extract in 10 ml distilled water); a portion of 2 ml + 2 ml FeCl_3_; blue-black precipitate indicated the presence of tannins. For saponins (frothing test: 0.5 ml filtrate + 5 ml distilled water); frothing persistence indicated presence of saponins. For steroids and triterpenoids (Liebermann-Burchard reaction: 5 mg plant extract in 10 ml chloroform, filtered); a 2 ml filtrate + 2 ml acetic anhydride + conc. H_2_SO_4_. Blue-green ring or pink-purple indicated the presence of steroids or triterpenoids. For flavonoids (5 mg plant extract in 10 ml methanol); a portion of 2 ml + conc. HCl + magnesium; ribbon pink-tomato red color indicated the presence of flavonoids. For anthocyanins (5 mg plant extract in 10 ml methanol); a portion 2 ml + 1%HCl +heating; orange color indicated the presence of anthocyanins. For anthraquinones (5 mg plant extract in 10 ml methanol); a portion of 2 ml + 2 ml ether-chloroform 1:1 v/v + 4 ml NaOH 10% (w/v); red color indicated the presence of anthraquinones. For phenols (5 mg plant material in 10 ml methanol); a portion of 2 ml + 2 ml FeCl_3_; violet-blue or greenish color indicated the presence of phenols.

### Chemicals for antimicrobial assays

Tetracycline (TET), cefepime (FEP), streptomycin (STR), ciprofloxacin (CIP), norfloxacin (NOR), chloramphenicol (CHL), cloxacillin (CLX), ampicillin (AMP), erythromycin (ERY), kanamycin (KAN) (Sigma-Aldrich, St Quentin Fallavier, France) were used as reference antibiotics. *p*-Iodonitrotetrazolium chloride (INT) and phenylalanine arginine *β*-naphthylamide (PAßN) were used as microbial growth indicator and efflux pumps inhibitor respectively.

### Bacterial strains and culture media

The studied microorganisms included reference (from the American Type Culture Collection) and clinical (Laboratory collection) strains of *Providencia stuartii, Pseudomonas aeruginosa, Klebsiella pneumoniae, Escherichia coli, Enterobacter aerogenes *and *Enterobacter cloacae *(Table 2). They were maintained on agar slant at 4°C and sub-cultured on a fresh appropriate agar plates 24 h prior to any antimicrobial test. Mueller Hinton Agar was used for the activation of bacteria. The Mueller Hinton Broth (MHB) was used for the MIC determinations.

**Table 2:** Bacterial strains and features

Strains	Features	References
***Escherichia coli***		
ATCC8739 and ATCC10536	Reference strains	
AG100	Wild-type *E. coli *K-12	[39]
AG100A	AG100 *ΔacrAB*::KAN^R^	[39]
AG100A_TET_	Δ*acrAB *mutant de AG100, avec le gène *acrF *sur-exprimé; TET^R^	[39]
AG102	Δ*acrAB *mutant AG100, owing *acrF *gene markedly over-expressed; TET^R^	[40]
MC4100	Wild type *E. coli*	
W311O	Wild type *E. coli*	[41]
***Enterobacter aerogenes***		
ATCC13048	Reference strains	
EA-CM64	CHL^R ^resistant variant obtained from ATCC13048 over-expressing the AcrAB pump	[42]
EA3	Clinical MDR isolate; CHL^R^, NOR^R^, OFX^R^, SPX^R^, MOX^R^, CFT^R^, ATM^R^, FEP^R^	[42]
EA27	Clinical MDR isolate exhibiting energy-dependent norfloxacin and chloramphenicol efflux with KAN^R ^AMP^R ^NAL^R ^STR^R ^TET^R^	[42,43]
EA289	KAN sensitive derivative of EA27	[43,44]
EA294	EA289 a*crA::*KAN^R^	[44]
EA298	EA 289 *tolC::*KAN^R^	[44]
***Enterobacter cloacae***		
ECCI69	Clinical isolates	Laboratory collection of UNR-MD1, University of Marseille, France
BM47	Clinical isolates	Laboratory collection of UNR-MD1, University of Marseille, France
BM67	Clinical isolates	Laboratory collection of UNR-MD1, University of Marseille, France
***Klebsiella pneumoniae***		
ATCC12296	Reference strains	
KP55	Clinical MDR isolate, TET^R ^, AMP^R^, ATM^R^, CEF^R^	[45]
KP63	Clinical MDR isolate, TET^R^, CHL^R^, AMP^R^, ATM^R^	[45]
K24	AcrAB-TolC	Laboratory collection of UNR-MD1, University of Marseille, France
K2	AcrAB-TolC	Laboratory collection of UNR-MD1, University of Marseille, France
***Providencia stuartii***		
NEA16	Clinical MDR isolate, AcrAB-TolC	
ATCC29914	Clinical MDR isolate, AcrAB-TolC	[46]
PS2636	Clinical MDR isolate, AcrAB-TolC	
PS299645	Clinical MDR isolate, AcrAB-TolC	
***Pseudemonas aeruginosa***		
PA 01	Reference strains	
PA 124	MDR clinical isolate	[26]

^a^AMP, ATM^R^, CEF^R^, CFT^R^, CHL^R^, FEP^R^, KAN^R^, MOX^R^, STR^R^, TET^R^. Resistance to ampicillin, aztreonam, cephalothin, cefadroxil, chloramphenicol, cefepime, kanamycin, moxalactam, streptomycin, and tetracycline; MDR: Multidrug resistant.

### Bacterial susceptibility determinations

The respective MICs of samples on the studied bacteria were determined using rapid INT colorimetric assay [6,7]. Briefly, the test samples were first dissolved in DMSO/MHB. The solution obtained was then added to MHB, and serially diluted two fold (in a 96-wells microplate). One hundred microlitres (100 μl) of inoculum (1.5 × 10^6 ^CFU/ml) prepared in MHB was then added. The plates were covered with a sterile plate sealer, then agitated to mix the contents of the wells using a shaker and incubated at 37°C for 18 h. The final concentration of DMSO was lower than 2.5% and did not affect the microbial growth. Wells containing MHB, 100 μl of inoculum and DMSO at a final concentration of 2.5% served as negative control (this internal control was systematically added). The total volume in each well was 200 μl. Chloramphenicol was used as reference antibiotic. The MICs of samples were detected after 18 h incubation at 37°C, following addition (40 μl) of 0.2 mg/ml INT and incubation at 37°C for 30 minutes. Viable bacteria reduced the yellow dye to pink. MIC was defined as the lowest sample concentration that prevented this change and exhibited complete inhibition of microbial growth [8].

Samples were tested alone and then, in the presence of PAßN at 30 μg/ml final concentration. Two of the best extracts [those from *D. glomerata and B. cinnamomea*] were also tested in association with antibiotics at MIC/2 and MIC/5. These concentrations were selected following a preliminary assay on one of the tested MDR bacteria, *P. aeruginosa *PA124 (See Additional file 1, Table A1). All assays were performed in triplicate and repeated thrice. Fractional inhibitory concentration (FIC) was calculated as the ratio of MIC_Antibiotic in combination_/MIC_Antibiotic alone _and the interpretation made as follows: synergistic (*<*0.5), indifferent (0.5 to 4), or antagonistic (*>*4) [9] (The FIC values available in Additional file 1, Tables A2 and A3).

## Results

### Phytochemical composition of the spice extracts

The results of the phytochemical studies (Table 3) showed that all the tested extracts contain alkaloids, phenols and tannins. Anthocyanins, anthraquinones, flavonoids, saponins, sterols and triterpenes were selectively present.

**Table 3:** Extraction yields, aspects and phytochemical composition of the plant extracts.

Spice samples	Extraction yield*	Physical aspect	Phytochemical composition								
			
			Alkaloids	Anthocyanins	Anthraquinones	Flavonoids	Phenols	Saponins	Tannins	Sterols	Triterpenes
*Fagara xanthoxyloides*	12.13	Oily brown	+	-	+	+	+	-	+	-	-
*Dichrostachys glomerata*	18.29	Brown paste	+	+	+	+	+	+	+	+	+
*Aframomum citratum*	16.32	Brown paste	+	-	-	+	+	+	+	+	-
*Beilschmiedia cinnamomea*	5.67	Black paste	+	+	+	+	+	-	+	-	+
*Echinops giganteus*	8.87	Oily brown	+	+	+	+	+	-	+	-	+
*Mondia whitei*	7.33	Brown paste	+	+	+	+	+	+	+	+	+
*Olax subscorpioidea*	12.34	Brown paste	+	-	+	+	+	-	+	-	+
*Solanum melongena*	14.30	Black paste	+	+	+	+	+	+	+	+	+
*Piper capense*	12.87	Brown paste	+	-	-	-	+	+	+	+	+
*Xylopia aethiopica*	26.42	Brown paste	+	-	-	-	+	+	+	-	+
*Scorodophloeus zenkeri*	4.67	Brown paste	+	-	-	-	+	+	+	-	+

(+): Present; (-): Absent; *The yield was calculated as the ratio of the obtained methanol extract according to the initial mass of the spice powder

### Antibacterial activity of the spice extracts

The results of the antibacterial activity of the extract alone on a panel of Gram negative bacteria are summarized in Table 4. It appears that the extract from *D. glomerata *was able to prevent the growth of all the twenty nine tested bacteria with MIC ≤ 1024 μg/ml. All other samples showed selective activity; their inhibitory activity being recorded on 28 of the 29 (96.6%) tested bacteria for *B. cinnamomea*, 24/29 (82.8%) for *A. citratum*, 19/29 (62.5%) for *P. capense*, 18/29 (62.1%) for *E. giganteus *and *F. Xanthoxyloïdes*, 15/29 (51.7%) for *O. subscorpioïdea*, 13/29 (44.8%) for *X. aethiopica*, 12/29 (41.4%) for *M. whitei*, 6/29 (20.7%) for *S. melongena *and 4/29 (13, 79%) for *S. zenkeri*.

**Table 4:** Minimal inhibitory concentration (MIC) of the studied spice extracts and CHL on the studied bacterial species.

	Tested samples and MIC in μg/ml in the absence and presence of PAßN (in parenthesis)											
	
Bacterial strains	*F*. *xanthoxyloides*	*D*. *glomerata*	*A*. *citratum*	*B. cinnamomea*	*E*. *giganteus*	*M*. *whitei*	*S*. *melongena*	*O*. *subscorpioidea*	*P*. *capense*	*X*. *aethiopica*	*S. zenkeri*	CHL
***E. coli***												
ATCC8739	- (1024)	1024 (512)	1024 (256)	- (256)	- (-)	- (1024)	- (128)	- (-)	- (512)	- (512)	- (-)	4 (< 2)
ATCC10536	1024	512 (128)	1024	1024	1024	1024	-	512	256	64	256	< 2 (< 2)
AG100	256	512 (256)	1024 (1024)	1024 (1024)	1024 (1024)	1024 (-)	1024 (-)	512 (-)	- (1024)	1024 (1024)	- (-)	8 (< 2)
AG100A	1024	1024 (256)	1024	1024	1024	-	-	1024	1024	-	-	< 2 (< 2)
AG100A_TET_	-	1024 (512)	1024	512	1024	-	-	-	1024	1024	-	64 (< 2)
AG102	1024	512 (256)	1024	512	1024	-	-	1024	1024	1024	-	32 (< 2)
MC4100	256	256(< 8)	512	256	512	1024	1024	1024	1024	1024	-	32
W3110	- (512)	256 (< 8)	512 (< 8)	1024 (< 8)	1024 (1024)	1024 (512)	-	512 (512)	1024 (1024)	- (-)	- (-)	4 (< 2)
***E. aerogenes***	-											
ATCC13048	-	512 (512)	1024	512	-	-	-	-	-	-	-	8 (< 2)
CM64	- (-)	512 (512)	- (1024)	1024 (1024)	1024 (1024)	1024 (-)	- (-)	- (-)	- (-)	- (-)	- (-)	256 (8)
EA3	1024	1024 (1024)	-	1024	-	-	-	1024	1024	-	-	- (128)
EA27	1024	512 (256)	1024	1024	1024	1024	-	512	1024	1024	-	256 (< 2)
EA289	-	1024 (1024)	1024	512	1024	-	-	-	-	-	-	- (64)
EA298	-	1024 (128)	-	1024	-	1024	-	256	512	1024	128	64 (< 2)
EA294	256	128	512	64	256	-	1024	256	256	256	512	8
***E. cloacae***												
ECCI69	512 (-)	1024 (1024)	1024 (1024)	1024 (512)	1024 (-)	- (-)	- (-)	- (-)	-	-(1024)	- (256)	- (16)
BM47	1024 (1024)	1024 (256)	1024 (1024)	1024 (1024)	1024 (-)	- (-)	- (-)	- (-)	512(64)	1024(1024)	- (-)	- (< 2)
BM67	1024 (1024)	1024 (128)	1024	1024	-	-	-	-	512	1024	-	256 (16)
***K. pneumoniae***												
ATCC11296	1024 (1024)	512 (128)	512 (512)	256 (256)	1024 (256)	- (1024)	- (1024)	1024 (512)	1024 (256)	- (-)	- (-)	4 (< 2)
KP55	1024	512 (256)	1024	512	-	-	-	1024	1024	-	-	64 (2)
KP63	1024	512 (< 8)	256	512	512	1024	512	256	256	64	-	64 (< 2)
K24	512	512 (32)	512	256	32	1024	1024	-	1024	-	-	16 (< 2)
K2	-	1024 (128)	-	1024	-	-	-	1024	512	-	-	32 (4)
***P. stuartuii***												
NEA16	1024	512 (32)	128	256	1024	1024	1024	512	256	512	1024	32 (8)
ATCC29914	1024	1024 (512)	512	512	1024	1024	-	-	1024	-	-	16 (8)
PS2636	-	512	1024	1024	-	-	-	-	-	-	-	32
PS299645	1024 (-)	256 (256)	256	256 (512)	- (-)	- (-)	- (512)	- (-)	- (-)	- (-)	- (-)	32 (< 2)
***P. aeruginosa***												
PA01	-	1024 (512)	1024	1024	-	-	-	-	-	1024	-	16 (< 2)
PA124	-	512 (512)	-	1024	-	1024	-	-	-	-	-	32 (< 2)

(**-**): MIC not detected at up to 1024 μg/ml for the les extracts and 256 μg/ml for CHL. **()**: values in parenthesis are MIC of substance in the presence of PAßN at 30 μg/ml. The MIC of PAßN was 64 μg/ml on *E. coli*, AG100A, 512 μg/ml on ATCC11296, BM67, EA27, EA289; 1024 μg/ml on AG100A_TET_, ATCC13048, CM64; and > 1024 μg/ml on other bacteria. CHL: chloramphénicol

MIC values below 100 μg/ml (Table 4) were recorded with the extract of *B. cinnamomea *against *Enterobacter aerogenes *EA294 (64 μg/ml), *E. giganteus *on *Klebsiella pneumoniae *K24 (32 μg/ml) and *X. aethiopica *on *Escherichia coli *ATCC10536 and *Klebsiella pneumoniae *KP63 (64 μg/ml).

### Role of efflux pumps in susceptibility of Gram negative bacteria to the tested spice extracts

The various strains and MDR isolates were tested for their susceptibilities to the spice extracts, and reference antibiotic, CHL in the presence of PAßN, a well-known efflux pump inhibitor. The results presented in Table 4 showed that the activity of the extract from *D. glomerata *significantly increased in the presence of PAßN on 18/26 (69.2%) of the tested bacteria. The MIC values below 100 μg/ml were noted with this extract against *E. coli *MC4100 and W3110 (< 8 μg/ml), *K. pneumoniae *KP63 and K24 (< 8 μg/ml and 32 μg/ml respectively) and *P. stuartii *NAE16 (32 μg/ml). Apart from the extract of *D. glomerata*, PAßN did not induce an increased activity of other tested extract.

### Effects of the association of some spice extracts with antibiotics

To evaluate the possible synergistic effects of the extracts with antibiotics, four of the most active samples (*F. xanthoxyloïdes, D. glomerata, B. cinnamomea *and *O. subscorpioïdea*) were selected. A preliminary study using *P. aeruginosa *PA124, one of the MDR bacteria used in this work, was carried out with ten antibiotics (CLX, AMP, ERY, KAN, CHL, TET, FEP, STR, CIP and NOR) to select the appropriate sub-inhibitory concentrations to be used. The results (see Additional file 1, Table A1) allow the selection of MIC/2 and MIC/5 as the sub-inhibitory concentrations of the extracts from *D. glomerata *and *B. cinnamomea*, which were then tested on eight MDR bacteria, *E. coli *AG100, AG100_TET_, *K. pneumoniae *KP55, *E. aerogenes *EA3, EA27, EA289, CM64 in addition to *P. aeruginosa *PA124. The results are summarized in Tables 5 and 6. Synergistic effects were observed with the association between *D. glomerata *(Table 5, Additional file 1, Table A2) and *B. cinnamomea (*Table 6, Additional file 1, Table A3) and most of the antibiotics on the studied MDR bacteria. At MIC/2, synergistic effects were noted with the extract of *D. glomerata *on 25% (2/8) of the tested bacteria for CLX and AMP, 50% (4/8) for KAN, 62.5% (5/8) for CHL, FEP, STR, CIP, 75% (6/8) for ERY and 87.5% (7/8) for NOR and TET. Increase in MIC values of > 8 fold were recorded at MIC/2 with CHL, TET, STR, CIP, NOR (Table 5). At MIC/5, synergistic effects were noted on 50% of the eight tested MDR bacteria in the case of STR and CIP, 62.5% in the case of ERY and 75% in the case of CHL, TET and NOR.

**Table 5:** Minimal inhibitory concentration (MIC) in μg/ml of antibiotics in the absence and presence of the sub-inhibitory concentrations of *D. glomerata *extracts against MDR bacteria.

Bacterial strains	Antibiotics and MIC in absence and presence *D. glomerata *extracts at MIC/2 and MIC/5														
	
	Chloramphenicol			Cloxacillin			Ampicillin			Erythromycin			Kanamycin		
	
	Alone	MIC/2	MIC/5	Alone	MIC/2	MIC/5	Alone	MIC/2	MIC/5	Alone	MIC/2	MIC/5	Alone	MIC/2	MIC/5
PA124	32	32 (1) ^**I**^	16 (2) ^**s**^	-	-	-	64	-	-	64	32 (2) ^**s**^	64 (1) ^**I**^	64	16 (4) ^**s**^	64 (1)
CM64	256	256 (1) ^**I**^	-	-	-	-	-	-	256	-	64	128	1	1 (1) ^**I**^	1(1) ^**I**^
EA3	-	32 (> 16) ^**s**^	32 (> 16) ^**s**^	-	256	256	-	-	-	64	32 (2) ^**s**^	64 (1) ^**I**^	32	16 (2) ^**s**^	16 (2) ^**s**^
EA27	256	32 (8) ^**s**^	64 (4) ^**s**^	256	64 (4) ^**s**^	64 (4) ^**s**^	64	64 (1) ^**I**^	64 (1) ^**I**^	32	32 (1) ^**I**^	32 (1) ^**I**^	16	16 (1) ^**I**^	16 (1) ^**I**^
EA289	-	-	-	-	-	-	-	256	64	256	64 (4) ^**s**^	128 (2) ^**s**^	4	< 2 (> 2) ^**s**^	4 (1) ^**I**^
KP55	64	8 (8) ^**s**^	16 (4) ^**s**^	-	-	-	-	-	-	256	128 (2) ^**s**^	128 (2) ^**s**^	32	32 (1) ^**I**^	32 (1) ^**I**^
AG100A_TET_	64	8 (8) ^**s**^	16 (4) ^**s**^	-	-	-	-	16	32	32	32 (1) ^**I**^	16 (2) ^**s**^	32	2 (16) ^**s**^	8 (4) ^**s**^
AG100	8	< 2 (> 4) ^**s**^	< 2 (> 4) ^**s**^	256	128 (2) ^**s**^	64 (4) ^**s**^	64	4 (16) ^**s**^	4 (16) ^**s**^	32	< 2 (> 16) ^**s**^	4 (8) ^**s**^	< 2	< 2	< 2
**Bacterial strains**	**Tetracycline**			**Cefepime**			**Streptomycin**			**Ciprofloxacin**			**Norfloxacin**		
	
	**Alone**	**MIC/2**	**MIC/5**	**Alone**	**MIC/2**	**MIC/5**	**Alone**	**MIC/2**	**MIC/5**	**Alone**	**MIC/2**	**MIC/5**	**Alone**	**MIC/2**	**MIC/5**
PA124	4	< 0, 5 (> 8) ^**s**^	2 (2) ^**s**^	-	-	-	16	16 (1) ^**I**^	16 (1) ^**I**^	16	< 0, 5 (> 8) ^**s**^	16 (1) ^**I**^	128	-	-
CM64	32	4 (8) ^**s**^	8 (4) ^**s**^	256	64 (4) ^**s**^	128 (2) ^**s**^	8	< 2 (> 4) ^**s**^	4 (2) ^**s**^	1	1 (1) ^**I**^	1 (1) ^**I**^	2	1 (2) ^**s**^	1 (2) ^**s**^
EA3	2	1 (2) ^**s**^	2 (2) ^**s**^	-	-	-	16	8 (2) ^**s**^	8 (2) ^**s**^	64	4 (16) ^**s**^	64 (1) ^**I**^	64	32 (2) ^**s**^	64 (1) ^**I**^
EA27	16	4 (4) ^**s**^	8 (2) ^**s**^	256	128 (2) ^**s**^	128 (2) ^**s**^	8	4 (2) ^**s**^	4 (2) ^**s**^	2	2 (1) ^**I**^	2 (1) ^**I**^	16	2 (8) ^**s**^	4 (4) ^**s**^
EA289	8	1 (8) ^**s**^	2 (4) ^**s**^	-	256 (> 2) ^**s**^	-	64	8 (8) ^**s**^	32 (2) ^**s**^	32	16 (2) ^**s**^	16 (2) ^**s**^	64	16 (4) ^**s**^	32 (2) ^**s**^
KP55	4	2 (2) ^**s**^	2 (2) ^**s**^	-	128 (> 4) ^**s**^	-	8	8 (1) ^**I**^	8 (1) ^**I**^	128	4 (32) ^**s**^	32 (4) ^**s**^	128	32 (4) ^**s**^	32 (4) ^**s**^
AG100A_TET_	4	1 (4) ^**s**^	2 (2) ^**s**^	-	32(> 16) ^**s**^	-	16	2 (8) ^**s**^	16 (1) ^**I**^	64	32 (2) ^**s**^	16 (4) ^**s**^	64	8 (8) ^**s**^	16 (4) ^**s**^
AG100	< 2	< 2 (> 4) ^**s**^	< 2 (> 4) ^**s**^	256	< 2 (> 128) ^**s**^	< 2 (> 128) ^**s**^	256	256 (1) ^**I**^	256 (1) ^**I**^	< 2	< 2	< 2	32	4 (8) ^**s**^	32 (1) ^**I**^

MIC/2: concentration of plant extract added equal to 256 μg/ml for PA124, CM64, EA3, EA27, KP55, AG100; and to 512 μg/ml for EA289 and AG100A_TET_.

MIC/5: concentration of plant extract added equal to 102.4 μg/ml for PA124, CM64, EA3, EA27, KP55, AG100; and to 204.8 μg/ml for EA289 and AG100A_TET_

(): Values in bracket are folds increase of activity. S: synergy, I: indifference; (-): > 512 μg/ml

**Table 6:** Minimal inhibitory concentration (MIC) of antibiotics in the absence and presence of the sub-inhibitory concentrations of *B. cinnamomea *extract (μg/ml) against some MDR bacteria.

Bacterial strains	Antibiotics and MIC in absence and presence *B. cinnamomea *extracts at MIC/2 and MIC/5														
	
	Chloramphenicol			Cloxacillin			Ampicillin			Erythromycin			Kanamycin		
	
	Alone	MIC/2	MIC/5	Alone	MIC/2	MIC/5	Alone	MIC/2	MIC/5	Alone	MIC/2	MIC/5	Alone	MIC/2	MIC/5
PA124	32	< 0, 5 (> 32) ^**s**^	32 (1) ^**s**^	-	-	-	-	-	-	64	8 (8) ^**s**^	32 (2) ^**s**^	32	32 (1)	64
CM64	256	-	-	-	-	-	-	-	-	-	64 (> 8) ^**s**^	64 (> 8) ^**s**^	1	< 0.5 (> 2) ^**s**^	< 0.5 (> 2) ^**s**^
EA3	-	16 (> 32) ^S^	32	-	256	-	-	-	-	64	8 (8) ^**s**^	32 (2) ^**s**^	32	16 (2) ^**s**^	16 (2) ^**s**^
EA27	256	8 (32) ^**s**^	16 (16) ^**s**^	256	< 0, 5 (> 512) ^**s**^	16(16) ^**s**^	64	64 (1) ^**I**^	64 (1) ^**I**^	32	8 (4) ^**s**^	32 (2) ^**s**^	16	< 0.5 (> 32) ^**s**^	16(1) ^**I**^
EA289	-	256	-	-	-	-	-	-	-	256	32 (8) ^**s**^	64 (4) ^**s**^	4	4 (1) ^**I**^	4 (1) ^**I**^
KP55	64	8 (8) ^**s**^	8 (8) ^**s**^	-	-	-	-	-	-	256	128 (2) ^**s**^	128 (2) ^**s**^	32	32 (1) ^**I**^	32 (1) ^**I**^
AG100A_TET_	64	16 (4) ^**s**^	32 (2) ^**s**^	-	128 (> 4) ^S^	128 (> 4) ^S^	-	8 (> 64) ^S^	32 (16) ^S^	32	32 (1) ^**I**^	32 (1) ^**I**^	32	1 (32) ^**s**^	8 (4) ^**s**^
AG100	8	< 2 (> 4) ^**s**^	4 (2) ^**s**^	256	256 (1)	256 (1)	64	< 2(> 32) ^**s**^	4 (16) ^**s**^	32	< 2 (> 16) ^**s**^	4 (8) ^**s**^	< 2	< 2	< 2

**Bacterial strains**	**Tetracycline**			**Cefepime**			**Streptomycin**			**Ciprofloxacin**			**Norfloxacin**		
	
	**Alone**	**MIC/2**	**MIC/5**	**Alone**	**MIC/2**	**MIC/5**	**Alone**	**MIC/2**	**MIC/5**	**Alone**	**MIC/2**	**MIC/5**	**Alone**	**MIC/2**	**MIC/5**
	
PA124	4	2 (2) ^**s**^	4 (1) ^**s**^	-	64 (> 8) ^**s**^	-	16	16 (1)	16 (1)	16	16 (1) ^**I**^	16 (1) ^**I**^	128	64 (2) ^**s**^	64 (2) ^**s**^
CM64	32	8 (4) ^**s**^	8 (4) ^**s**^	256	32 (8) ^**s**^	32 (8) ^**s**^	8	< 2 (> 4) ^**s**^	< 2 (> 4) ^**s**^	1	1 (1) ^**I**^	1 (1) ^**I**^	2	1 (2) ^**s**^	2 (1) ^**I**^
EA3	2	1 (2) ^**s**^	2 (1) ^**I**^	-	-	-	16	4 (4) ^**s**^	4 (4) ^**s**^	64	4 (16) ^**s**^	8(8) ^**s**^	64	16 (4) ^**s**^	32 (2) ^**s**^
EA27	16	1 (16) ^**s**^	4 (4) ^**s**^	256	16 (16) ^**s**^	64(4) ^**s**^	8	< 0.5 (> 16) ^**s**^	2 (4) ^**s**^	2	< 0.5 (> 4) ^**s**^	2 (1) ^**I**^	16	< 0.5 (> 32) ^**s**^	4 (4) ^**s**^
EA289	8	4 (2) ^**s**^	4 (2) ^**s**^	-	256 (> 2) ^**s**^	256 (> 2) ^**s**^	64	16 (4) ^**s**^	64 (1) ^**I**^	32	16 (2) ^**s**^	16 (2) ^**s**^	64	32 (2) ^**s**^	32 (2) ^**s**^
KP55	4	4 (1) ^**I**^	4 (1) ^**I**^	-	64 (> 8) ^**s**^	128 (> 2) ^**s**^	8	8 (1) ^**I**^	8 (1) ^**I**^	128	8 (16) ^**s**^	16 (8) ^**s**^	128	16 (8) ^**s**^	32 (4) ^**s**^
AG100A_TET_	4	2 (2) ^**s**^	2 (2) ^**s**^	-	256 (> 2) ^**s**^	64(> 4) ^**s**^	16	8 (2) ^**s**^	16 (1) ^**I**^	64	4 (16) ^**s**^	8 (8) ^**s**^	64	32 (2) ^**s**^	64 (1) ^**I**^
AG100	< 2	< 2	< 2	256	64 (4) ^**s**^	64 (4) ^**s**^	256	256 (1) ^**I**^	256 (1) ^**I**^	4	< 2 (> 2) ^**s**^	< 2 (> 2) ^**s**^	32	4 (8) ^**s**^	32 (1) ^**I**^

MIC/2: concentration of plant extract added equal to 512 μg/ml for PA124, CM64, EA3, EA27, AG100; and to 256 μg/ml for EA289, KP55 and AG100A_TET_

MIC/5: concentration of plant extract added equal to 204.8 μg/ml for PA124, CM64, EA3, EA27, KP55, AG100; and to102.4 μg/ml for EA289 and AG100A_TET_

(): Values in bracket are folds increase of activity. S: synergy, I: indifference; (-): > 512 μg/ml

The extract of *B. cinnamomea *at MIC/2 (Table 6) also induced significant increase of the activity of several antibiotics, the synergistic effects being noted on 25% of the tested bacteria in the case of CLX and AMP, 50% in the case of KAN, 62.5% in the case of FEP and STR, 75% in the case of CHL, TET and CIP, 87.5% in the case of ERY and 100% for NOR. With this extract, synergistic effects were also observed at MIC/5 on 25% of the studied MDR bacteria in the case of CLX and AMP, 37.5% in the case of STR and KAN, 50% in the case of TET, 62.5% in the case of CHL, FEP, NOR and CIP and 75% in the case of ERY.

## Discussion

### Phytochemical composition of the spice extracts

Phytochemical screening revealed the presence of several classes of secondary metabolites. Though the detection of such metabolites does not automatically predict the antimicrobial activity of a plant extract, it has clearly been demonstrated that several compounds belonging to the investigated classes of metabolites showed antibacterial activities [4,10-12].

### Antibacterial activity of the spice extract

Phytochemicals are routinely classified as antimicrobials on the basis of susceptibility tests that produce MIC in the range of 100 to 1000 μg/ml [13]. Activity is considered to be significant if MIC values are below 100 μg/ml for crude extract and moderate when 100 < MIC < 625 μg/ml [11]. Therefore, the activity recorded with *B. cinnamomea *and *E. giganteus *respectively on *E. aerogenes *EA294 and *K. pneumoniae *K24, and *X. aethiopica *on *E. coli *ATCC10536 and *K. pneumoniae *KP63 can be considered significant. Alternative criteria have been described by Fabry et al. [14], which consider extracts having MIC values below 8000 μg/ml to have noteworthy antimicrobial activity. Under these less stringent criteria, and considering the fact that the spices tested are used as food ingredients with limited toxicity, the overall activity recorded with several extracts, most notably those of *D. glomerata, B. cinnamomea*, *A. citratum*, *P. capense*, *E. giganteus, F. Xanthoxyloïdes *and *O. subscorpioïdea*, could be considered important. Besides, some of the tested samples were more active than CHL used as reference antibiotic on some of the MDR bacteria such as *E. cloacae *ECCI69 and BM47, *E. aerogenes *EA27 and EA289, highlighting the importance of the results reported herein. It can be noted that all the investigated phytochemical classes were detected in the extracts of *D. glomerata, S. melongena *and *M. withei*. Contrary to *D. glomerata *extract that exhibited a good spectrum of activity, the inhibition potential of *S. melongena *and *M. withei *was lower and seems not in correlation with their chemical composition. This clearly confirms the fact that the presence of secondary metabolites does not automatically predict the antimicrobial activity of a plant extract though it is a good indication of its possible pharmacological potential.

To the best of our knowledge, the antibacterial activity of *B. cinnamomea *and *P. capense *is being reported for the first time. Moreover, the present work reports for the first time the activity of the tested spices on MDR bacteria. Nevertheless, the antimicrobial potential of some of the plants or related genus were demonstrated on sensitive strains. Banso and Adeyemo [15] reported the presence of antibacterial tannins in the genus *Dichrostachys*. Chouna et al. [16] also demonstrated that *Beilschmiedia anacardioides *was significantly active against *Bacillus subtilis*, *Micrococcus luteus *and *Streptococcus faecalis*. Plants of the genus *Echinops *such as *E. ellenbeckii *and *E. longisetus *were found active on *Staphylococcus aureus *[17] meanwhile the antibacterial activity of the essential oils and alkaloids from *F. xanthoxyloïdes *was also documented [18,19]. The aqueous and ethanol extracts from *O. subscorpioïdea *were found active on both bacteria and fungi [20]. The results obtained in the present work therefore provide additional information on the studied plants and are in consistence with some of the previous reports.

### Role of efflux pumps in susceptibility of Gram negative bacteria to the tested spice extracts

Tripartite efflux systems, mainly those clinically described as AcrAB-TolC in *Enterobacteriaceae *or MexAB-OprM in *P. aeruginosa*, are associated with a major human health problem as they play a central role in multidrug resistance of pathogenic Gram negative bacteria [21-23]. PAßN has been reported as a potent inhibitor of the RND efflux systems and is especially active on AcrAB-TolC and MexAB-OprM [22,24,25]. To determine the role of efflux pumps in this work, the concentration of PAßN used (30 μg/ml) had no intrinsic effect on the bacteria as previously determined [26]. In contrast, with these conditions significant increase of the antibacterial activity of *D. glomerata *extract was noted, showing that one or more active compounds from this plant could be substrate(s) of efflux pumps acting in resistant strains of *E. coli, K. pneumoniae *and *P. stuartii*. These data suggest that possible association of the extract of *D. glomerata *and efflux pump inhibitor can be envisaged to improve the fight against MDR phenotypes.

### Effects of the association of extracts from *D. glomerata *and *B. cinnamomea *with antibiotics

The association of natural products such as plant extracts and antibiotics constitutes an alternative in the fight against MDR bacteria. Significant synergistic effects were noted with both *D. glomerata *and *B. cinnamomea *extracts when they were associated with several antibiotics. Such effects might be due either to the action of the active compounds or possible inhibition of the efflux pumps by other compounds of the extracts. The lowest synergistic effects were observed with *β*-lactamines (CLX and AMP), obviously due to the fact their target are localized in the bacterial cell coat. However, the synergistic effects observed indicate that active compounds of the extract could also present different mode(s) of action from those of the studied antibiotics.

## Conclusion

The overall results of the present work provide baseline information for the possible use of the studied spice extracts in the treatment of bacterial infections involving MDR phenotypes. In addition to these antibacterial activities, the data reported herein indicated that possible combinations of the extract of *D. glomerata *with an efflux pump inhibitor, and also the association of extract of this plant as well as that from *B. cinnamomea *with several antibiotics could be used in the control of bacterial infections involving MDR phenotypes.

## Competing interests

The authors declare that they have no competing interests.

## Authors' contributions

PAF carried out the study; VK designed the experiments and wrote the manuscript; VK, IKV, JRK and JMP supervised the work; VK and JMP provided the bacterial strains; All authors read and approved the final manuscript.

## Pre-publication history

The pre-publication history for this paper can be accessed here:

http://www.biomedcentral.com/1472-6882/11/104/prepub

## Supplementary Material

Additional file 1 **Table S1**. Activities of antibiotics in combination with the sub-inhibitory concentrations of some plants extracts on *Pseudomonas aeruginosa *PA124. **Table S 2**. Fractional Inhibitory Concentrations (FIC) of the association between antibiotics and extract of *D. glomerata *at MIC/2 and MIC/5 (μg/ml) against MDR bacteria. **Table S 3**. Fractional Inhibitory Concentrations (FIC) of association between antibiotics and extract of *B. cinnamomea *at MIC/2 and MIC/5 (μg/ml). Click here for file
