# Supplementary material for: Antibacterial activities of selected Cameroonian spices and their synergistic effects with antibiotics against multidrug-resistant phenotypes
Source: BMC Complement Altern Med. 2011 Nov 1;11:104. doi: 10.1186/1472-6882-11-104 (PMC3228721; doi:10.1186/1472-6882-11-104)
Supplement: Additional file 1 — Table S1. Activities of antibiotics in combination with the sub-inhibitory concentrations of some plants extracts on Pseudomonas aeruginosa PA124. Table S 2. Fractional Inhibitory Concentrations (FIC) of the association between antibiotics and extract of D. glomerata at MIC/2 and MIC/5 (μg/ml) against MDR bacteria. Table S 3. Fractional Inhibitory Concentrations (FIC) of association between antibiotics and extract of B. cinnamomea at MIC/2 and MIC/5 (μg/ml). [file 1472-6882-11-104-S1.DOC]

**Additional file 1.**

| **Extract and concentrations** | | **Antibiotics and FIC** | | | | | | | | | | | | | | | | | | | |
| --- | --- | --- | --- | --- | --- | --- | --- | --- | --- | --- | --- | --- | --- | --- | --- | --- | --- | --- | --- | --- | --- |
|  |  | **CHL** | | **CLX** | | **AMP** | | **ERY** | | **KAN** | | **TET** | | **FEP** | | **STR** | | **CIP** | | **NOR** | |
|  | Alone | **32** | | **-** | | **-** | | **128** | | **64** | | **8** | | **-** | | **16** | | **16** | | **128** | |
| *Fagara*  *xanthoxyloides* | MIC | | **16 (0.5) ^s^** | | - (-) | | - (-) | | **64 (0.5) ^s^** | | **4** (**0.062) ^s^** | | **4** (**0.5) ^s^** | | - (-) | | ND | | 16 (1) ^I^ | | 128 (1) ^I^ |
|  | MIC/2 | | **16 (0.5) ^s^** | | - (-) | | - (-) | | **64 (0.5) ^s^** | | **8 (0.125) ^s^** | | **4** (**0.5) ^s^** | | - (-) | | ND | | 16 (1) ^I^ | | 128 (1) ^I^ |
|  | MIC/5 | | 32 (1) ^I^ | | - (-) | | - (-) | | **64 (0.5) ^s^** | | **4** (**0.062) ^s^** | | **4** (**0.5) ^s^** | | - (-) | | ND | | 16 (1) ^I^ | | 128 (1) ^I^ |
|  | MIC/10 | | **16 (0.5) ^s^** | | - (-) | | - (-) | | 128 (1) ^I^ | | **4** (**0.062) ^s^** | | 8 (1) ^I^ | | - (-) | | ND | | 16 (1) ^I^ | | 128 (1) ^I^ |
|  | MIC/20 | | **16 (0.5) ^s^** | | - (-) | | - (-) | | 128 (1) ^I^ | | **8 (0.125) ^s^** | | **4** (**0.5) ^s^** | | - (-) | | ND | | 16 (1) ^I^ | | 256 (2) ^I^ |
| *Dichrostachys*  *glomerata* | MIC | | **<0.5 (<0.016) ^s^** | | **<0.5 (0.002) ^s^** | | **<0.5 (0.002) ^s^** | | **<0.5 (<0.004) ^s^** | | **<0.5(<0.008) ^s^** | | **<0.5** (**0.062**) **^s^** | | **<0.5 (0.002) ^s^** | | **<0.5 (0.031)^s^** | | **<0.5 (<0.5) ^s^** | | **0.5 (<0.004) ^s^** |
|  | MIC/2 | | 32 (1) ^I^ | | - (-) | | - (-) | | 256 (2) ^I^ | | **32 (0.5) ^s^** | | **<0.5** (**0.062**) **^s^** | | - (-) | | 16 (1) ^I^ | | **<0.5 (<0.5) ^s^** | | - (-) |
|  | MIC/5 | | **16 (0.5) ^s^** | | - (-) | | - (-) | | 256 (2) ^I^ | | **32 (0.5) ^s^** | | **2 (0.25) ^s^** | | - (-) | | 16 (1) ^I^ | | 16 (1) ^I^ | | - (-) |
|  | MIC/10 | | 32 (1) ^I^ | | - (-) | | - (-) | | **64 (0.5) ^s^** | | **32 (0.5) ^s^** | | **4** (**0.5) ^s^** | | - (-) | | 16 (1) ^I^ | | 16 (1) ^I^ | | 128 (1) ^I^ |
|  | MIC/20 | | 32 (1) ^I^ | | - (-) | | - (-) | | 256 (2) ^I^ | | 64 (1) ^I^ | | 8 (1) ^I^ | | - (-) | | 16 (1) ^I^ | | 16 (1) ^I^ | | 128 (1) ^I^ |
| *Aframomum*  *citratum* | MIC | | **<4 ^s^** | | - (-) | | - (-) | | 128 (1) ^I^ | | **32 (0.5) ^s^** | | **<4 (<0.5) ^s^** | | - (-) | | **2 ^s^** | | **8 (0.5) ^s^** | | **16 (0.125) ^s^** |
|  | MIC/2 | | 64 (2) ^I^ | | - (-) | | - (-) | | 128 (1) ^I^ | | **32 (0.5) ^s^** | | **<4 (<0.5) ^s^** | | - (-) | | 16 (1) ^I^ | | 16 (1) ^I^ | | **32 (0.25) ^s^** |
|  | MIC/5 | | 64 (2) ^I^ | | - (-) | | - (-) | | 128 (1) ^I^ | | **32 (0.5) ^s^** | | 8 (1) ^I^ | | - (-) | | 16 (1) ^I^ | | 16 (1) ^I^ | | **64 (0.5) ^s^** |
|  | MIC/10 | | 64 (2) ^I^ | | - (-) | | - (-) | | 128 (1) ^I^ | | **32 (0.5) ^s^** | | 8 (1) ^I^ | | - (-) | | 16 (1) ^I^ | | 16 (1) ^I^ | | **64 (0.5) ^s^** |
|  | MIC/20 | | 64 (2) ^I^ | | - (-) | | - (-) | | 128 (1) ^I^ | | 64 (1) ^I^ | | 8 (1) ^I^ | | - (-) | | 16 (1) ^I^ | | 16 (1) ^I^ | | **64 (0.5) ^s^** |
| *Beilschmiedia*  *cinnamomea* | MIC | | **<0.5 (<0.016) ^s^** | | **<0.5 (0.002) ^s^** | | **<0.5 (0.002) ^s^** | | **<0.5 (<0.004) ^s^** | | **<0.5(<0.008) ^s^** | | **<0.5** (**0.062**) **^s^** | | **<0.5 (0.002) ^s^** | | **<0.5 (0.031)^s^** | | **<0.5 (<0.5) ^s^** | | **0.5 (<0.004) ^s^** |
|  | MIC/2 | | **<0.5 (<0.016) ^s^** | | - (-) | | - (-) | | **8 (0.062) ^s^** | | **32 (0.5) ^s^** | | **2 (0.25) ^s^** | | **64 (<0.25) ^s^** | | 16 (1) ^I^ | | 16 (1) ^I^ | | **64 (0.5) ^s^** |
|  | MIC/5 | | 32 (1) ^I^ | | - (-) | | - (-) | | **32 (0.25) ^s^** | | 64 (1) ^I^ | | **2 (0.25) ^s^** | | - (-) | | 16 (1) ^I^ | | 16 (1) ^I^ | | **64 (0.5) ^s^** |
|  | MIC/10 | | 64 ^I^ | | - (-) | | - (-) | | **32 (0.25) ^s^** | | **32 (0.5) ^s^** | | 8 (1) ^I^ | | - (-) | | 16 (1) ^I^ | | 32 (2) ^I^ | | **64 (0.5) ^s^** |
|  | MIC/20 | | 32 (1) ^I^ | | - (-) | | - (-) | | **64 (0.5) ^s^** | | - | | **4** (**0.5) ^s^** | | - (-) | | 16 (1) ^I^ | | 32 (2) ^I^ | | **64 (0.5) ^s^** |
| *Olax*  *subscorpioidea* | MIC | | 32 (1) ^I^ | | - (-) | | - (-) | | 128 (1) ^I^ | | **32 (0.5) ^s^** | | **<4 (<0.5) ^s^** | | - (-) | | 16 (1) ^I^ | | 32 (2) ^I^ | | - (-) |
|  | MIC/2 | | 32 (1) ^I^ | | - (-) | | - (-) | | 128 (1) ^I^ | | **32 (0.5) ^s^** | | **<4 (<0.5) ^s^** | | - (-) | | 16 (1) ^I^ | | 32 (2) ^I^ | | - (-) |
|  | MIC/5 | | 32 (1) ^I^ | | - (-) | | - (-) | | 128 (1) ^I^ | | **32 (0.5) ^s^** | | 8 (1) ^I^ | | - (-) | | 32 (2) ^I^ | | 32 (2) ^I^ | | - (-) |
|  | MIC/10 | | 32 (1) ^I^ | | - (-) | | - (-) | | 128 (1) ^I^ | | **32 (0.5) ^s^** | | 8 (1) ^I^ | | - (-) | | 32 (2) ^I^ | | 16 (1) ^I^ | | **64 (0.5) ^s^** |
|  | MIC/20 | | 32 (1) ^I^ | | - (-) | | - (-) | | 128 (1) ^I^ | | 64 (1) ^I^ | | 8 (1) ^I^ | | - (-) | | 32 (2) ^I^ | | 16 (1) ^I^ | | **64 (0.5) ^s^** |

Table S1. Activities of antibiotics in combination with the sub-inhibitory concentrations of some plants extracts on *Pseudomonas aeruginosa* PA124.

AMP : Ampicillin. cefepime: FEP. CHL : chloramphenicol ; KAN : Kanamycin. NOR : norfloxacin. STR : Streptomycin. TET : tetracycline; CIP : ciprofloxacin. CLX : cloxacillin. ERY : erythromycin. S : synergy. I : indifférence. **( )**: FIC Values. ND : Non determined. (**-**): CMI. CMI* >256 µg /mL and Non determined Values of FIC

**Table S2. Fractional Inhibitory Concentrations (FIC) of the association between antibiotics and extract of *D. glomerata* at MIC/2 and MIC/5 (µg/ml) against MDR bacteria.**

| **Bactéries** | **Antibiotics and FIC** | | | | | | | | | | | | | |
| --- | --- | --- | --- | --- | --- | --- | --- | --- | --- | --- | --- | --- | --- | --- |
|  | **Chloramphenicol** | |  | **Cloxacillin** | |  | **Ampicillin** | |  | **Erythromycin** | |  | **Kanamycin** | |
|  | **MIC/2** | **MIC/5** |  | **MIC/2** | **MIC/5** |  | **MIC/2** | **MIC/5** |  | **MIC/2** | **MIC/5** |  | **MIC/2** | **MIC/5** |
| **PA124** | 1 | 0.5 |  | - | - |  | >4 | - |  | 0.5 | 1 |  | 0.25 | 0.5 |
| **CM64** | - | - |  | - | - |  | - | - |  | <0.25 | <0.5 |  | 1 | 1 |
| **EA3** | <0.125 | <0.125 |  | - | - |  | - | - |  | 0.5 | 1 |  | 0.5 | 0.5 |
| **EA27** | 0.125 | 0.25 |  | 0.25 | 0.25 |  | 1 | 1 |  | 1 | 1 |  | 1 | 1 |
| **EA289** | - | - |  | - | - |  | - | <0.25 |  | 0.25 | 0.5 |  | <0.5 | 1 |
| **KP55** | 0.125 | 0.25 |  | - | - |  | - | - |  | 0.5 | 0.5 |  | 1 | 1 |
| **AG100A_TET_** | 0.125 | 0.25 |  | - | - |  | <0.062 | <0.125 |  | 1 | 0.5 |  | 0.062 | 0.25 |
| **AG100** | <0.25 | 0.25 |  | 0.5 | 0.25 |  | 0.062 | 0.062 |  | <0.062 | 0.125 |  | - | - |
|  | | | | | | | | | | | | | | |
| **Bactéries** | **Tetracycline** | |  | **Cefepime** | |  | **Streptomycin** | |  | **Ciprofloxacin** | |  | **Norfloxacin** | |
|  | **MIC/2** | **MIC/5** |  | **MIC/2** | **MIC/5** |  | **MIC/2** | **MIC/5** |  | **MIC/2** | **MIC/5** |  | **MIC/2** | **MIC/5** |
| **PA124** | <0.125 | 0.5 |  | - | - |  | 1 | 1 |  | <0.031 | 1 |  | > 2 | >2 |
| **CM64** | 0.125 | 0.25 |  | 0.25 | 0.5 |  | <0.25 | 0.5 |  | 1 | 1 |  | 0.5 | 0.5 |
| **EA3** | 0.5 | 1 |  | - | - |  | 0.5 | 0.5 |  | 0.062 | 1 |  | 0.5 | 0.5 |
| **EA27** | 0.25 | 0.5 |  | 0.5 | 0.5 |  | 0.5 | 0.5 |  | 1 | 1 |  | 0.125 | 0.25 |
| **EA289** | 0.125 | 0.25 |  | - | - |  | 0.125 | 0.5 |  | 0.5 | 0.5 |  | 0.25 | 0.5 |
| **KP55** | 0.5 | 0.5 |  | <0.5 | - |  | 1 | 1 |  | 0.031 | 0.25 |  | 0.25 | 0.25 |
| **AG100A_TET_** | 0.25 | 0.5 |  | <0.125 | - |  | 0.125 | 1 |  | 0.5 | 0.25 |  | 0.125 | 0.25 |
| **AG100** | - | - |  | <0.008 | <0.008 |  | 1 | 1 |  | - | <0.5 |  | 0.125 | 0.25 |

(**-**): Non determined Values of FIC. There is synergy when FIC ≤ 0.5. indifference when 0.5<FIC < 4. and antagonism. when FIC>4.

| **Bacterial strains** | **Antibiotics and FIC** | | | | | | | | | | | | | |
| --- | --- | --- | --- | --- | --- | --- | --- | --- | --- | --- | --- | --- | --- | --- |
|  | **Chloramphenicol** | |  | **Cloxacillin** | |  | **Ampicillin** | |  | **Erythromycin** | |  | **Kanamycin** | |
|  | **MIC/2** | **MIC/5** |  | **MIC/2** | **MIC/5** |  | **MIC/2** | **MIC/5** |  | **MIC/2** | **MIC/5** |  | **MIC/2** | **MIC/5** |
| **PA124** | <0.016 | 1 |  | - | - |  | - | - |  | 0.125 | 0.5 |  | 1 | 2 |
| **CM64** |  |  |  | - | - |  | - | - |  | <0.25 | <0.25 |  | <0.5 | <0.25 |
| **EA3** | <0.062 | <0.125 |  | - | - |  | - | - |  | 0.125 | 0.5 |  | 0.5 | 0.5 |
| **EA27** | 0.031 | 0.062 |  | <0.002 | 0.062 |  | 1 | 1 |  | 0.25 | 1 |  | <0.031 | 1 |
| **EA289** | - | - |  | - | - |  | - | - |  | 0.125 | 0.25 |  | 1 | 1 |
| **KP55** | 0.125 | 0.125 |  | ­­- | - |  | - | - |  | 0.5 | 0.5 |  | 1 | 1 |
| **AG100A_TET_** | 0.25 | 0.5 |  | <0.5 | <0.5 |  | <0.031 | <0.125 |  | 1 | 1 |  | 0.0312 | 0.25 |
| **AG100** | <0.25 | 0.5 |  | 1 | 1 |  | <0.031 | <0.062 |  | <0.062 | 0.125 |  | - | - |
|  | | | | | | | | | | | | | | |
| **Bacterial strains** | **Tetracycline** | |  | **Cefepime** | |  | **Streptomycin** | |  | **Ciprofloxacin** | |  | **Norfloxacin** | |
|  | **MIC/2** | **MIC/5** |  | **MIC/2** | **MIC/5** |  | **MIC/2** | **MIC/5** |  | **MIC/2** | **MIC/5** |  | **MIC/2** | **MIC/5** |
| **PA124** | 0.5 | 1 |  | <0.25 | - |  | 1,255000000000000000000000000000000000000000000000000000000000000000000000000000000000000000000000000000000000000000000000000000 | 1 |  | 1 | 1 |  | 0.5 | 0.5 |
| **CM64** | 0.25 | 0.25 |  | 0.125 | 0.125 |  | ­<0.25 | <0.25 |  | 1 | 1 |  | 0.5 | 1 |
| **EA3** | 0.5 | 1 |  | - | - |  | 0.25 | 0.25 |  | 0.062 | 0.125 |  | 0.25 | 0.5 |
| **EA27** | 0.062 | 0.25 |  | 0.062 | 0.25 |  | <0.062 | 0.25 |  | <0.25 | 1 |  | <0.031 | 0.25 |
| **EA289** | 0.5 | 0.5 |  | - | - |  | 0.25 | 1 |  | 0.5 | 0.5 |  | 0.5 | 0.5 |
| **KP55** | 1 | 1 |  | <0.25 | <0.5 |  | 1 | 1 |  | 0.062 | 0.125 |  | 0.125 | 0.25 |
| **AG100A_TET_** | 0.5 | 0.5 |  | - | <0.25 |  | 0.5 | 1 |  | 0.062 | 0.125 |  | 0.5 | 1 |
| **AG100** | - | - |  | 0.25 | 0.25 |  | 1 | 1 |  | <0.5 | <0.5 |  | 0.125 | 1 |

**Table S3.** Fractional Inhibitory Concentrations (FIC) of association between antibiotics and extract of *B. cinnamomea* at MIC/2 and MIC/5 (µg/ml).

(**-**): Non determined Values of FIC. There is synergy when FIC ≤ 0.5. indifference when 0.5<FIC < 4. and antagonism. when FIC>4.
